# Variation in left and right coronary artery physiology in patients with severe aortic stenosis

**DOI:** 10.3389/fcvm.2023.1227532

**Published:** 2023-08-03

**Authors:** Muhammad Sabbah, Thomas Engstrøm, Niels Thue Olsen, Jacob Lønborg

**Affiliations:** ^1^ The Heart Center, Rigshospitalet, Copenhagen University Hospital, Copenhagen, Denmark; ^2^ Department of Biomedical Sciences, University of Copenhagen, Copenhagen, Denmark; ^3^ Department of Cardiology, Copenhagen University Hospital—Herlev and Gentofte, Gentofte, Denmark

**Keywords:** coronary flow reserve/methods, coronary physiology, aortic stenosis, thermodilution, microvascular

It is widely accepted that severe aortic valve stenosis (AS) can cause exhaustion of coronary flow reserve (CFR), leading to angina pectoris despite the absence of obstructive coronary artery disease. However, it is not established whether CFR is uniformly affected in the left and right coronary arteries. We addressed the differences in coronary physiology between the left anterior descending artery (LAD) and right coronary artery (RCA) in patients with severe AS before aortic valve replacement, by performing pair-wise comparisons of coronary flow reserve (CFR), fractional flow reserve (FFR), absolute hyperemic flow (*Q*), and minimal microvascular resistance (*R_μ_*) in the LAD and RCA using intracoronary bolus and continuous saline thermodilution techniques (1). The patients in this analysis represent a subset from a previously published cohort in which the physiology of both the LAD and RCA was evaluated (1). Wilcoxon's signed rank test and paired *t*-test were used to test the differences in medians and means, respectively. Pearson's correlation coefficient was used to test the relationship between the difference in RCA–LAD CFR and left ventricular mass. Two-tailed *p*-values <0.05 were considered significant. IBM SPSS Statistics for Windows, version 28 (IBM Corp., Armonk, NY, USA), was used for statistical analyses.

A total of 27 patients were included in this analysis, of which 25 had a right-dominant system and two had a co-dominant system. The mean age was 74 ± 8 years, 11 (41%) were women, and the left ventricular ejection fraction was 67 ± 9%. The aortic valve area was 0.72 ± 0.19 cm^2^ with mean and peak gradients of 56 ± 15 mmHg and 87 ± 24 mmHg, respectively. Left ventricular hypertrophy (LVH) was present in 61% of patients (2). CFR in the LAD was significantly lower than in the RCA (Table 1). Although FFR was lower in the LAD than that in the RCA, the difference in CFR between the LAD and RCA remained statistically significant when using CFR adjusted for epicardial pressure loss (i.e., CFR divided by FFR, Table 1). There was no correlation between the absolute RCA–LAD CFR difference and the left ventricular mass indexed to body surface area, *r* = −0.011, *p* = 0.97. The resting transit time was significantly shorter in the LAD compared with that in the RCA, whereas hyperemic transit times were not significantly different (Table 1). Interestingly, absolute hyperemic flow and minimal microvascular resistance in the LAD and RCA were not significantly different (Table 1).

**Table 1:** Paired measurements of coronary physiological indices in the left anterior descending and right coronary artery in patients with severe aortic stenosis.

	LAD	RCA	*p*-Value
CFR	2.5 (1.4–2.9)	4.1 (3.1–5.0)	<0.001
FFR	0.89 (0.84–0.92)	0.95 (0.93–0.97)	<0.001
CFR adjusted for FFR	2.7 (1.5–3.6)	4.3 (3.2–5.5)	<0.001
*T*_mn,rest_, s	0.56 ± 0.31	1.18 ± 0.69	<0.001
*T*_mn,hyperemia_, s	0.24 ± 0.11	0.29 ± 0.16	0.15
*Q*_hyperemia_, ml/min	267 ± 101	255 ± 130	0.75
*R_μ_*, WU	294 (203–367)	319 (262–451)	0.11

*T*_mn_, mean transit time; *Q*, absolute volumetric flow; *R_μ_*, absolute microvascular resistance; WU, wood units.

A total of 27 paired coronary physiological measurements for the LAD and RCA of patients with severe aortic valve stenosis. Data are shown as median and interquartile range or mean ± SD. CFR adjusted for FFR is calculated as CFR divided by FFR.

In healthy subjects, the coronary vascular bed is organized such that CFR is similar in the overall territory of all coronary arteries (3). The main finding of this study is that in severe AS, CFR is lower in the LAD compared with that in the RCA. This difference was not due to a difference in epicardial disease burden. Instead, our results indicate a higher resting flow in the LAD compared with that in the RCA, as shown once before with a Doppler-based approach (4). A possible explanation for this is that a proportion of RCA flow supplies the right ventricle which escapes the impact of AS, whereas the LAD exclusively supplies the pressure-loaded left ventricle and is thus taxed with a higher resting flow. In our previous work, we have shown that CFR in the LAD significantly increases 6 months after aortic valve replacement (5). Although changes in the RCA were not assessed in that study, it is conceivable that aortic valve replacement ultimately evens out the differences in CFR between the LAD and RCA found in this study.

In summary, CFR in the LAD is significantly lower than that in the RCA in patients with severe AS and right-dominant coronary systems. The difference is driven by a higher resting flow in the LAD compared with that in the RCA.

## Data Availability

The raw data supporting the conclusions of this article will be made available by the authors upon reasonable request.
